# 1,2-Bis{4-[1-(anthracen-9-ylmeth­yl)-1*H*-1,2,3-triazol-4-yl]phen­yl}-1,2-bis­[4,5-bis­(methyl­sulfan­yl)-1,3-dithiol-2-yl­idene]ethane

**DOI:** 10.1107/S1600536812045254

**Published:** 2012-11-07

**Authors:** Karimulla Mulla, Yuming Zhao, Louise N. Dawe

**Affiliations:** a Department of Chemistry, Memorial University of Newfoundland, St Johns, NL, Canada A1B 3X7; b Department of Chemistry and C-CART X-Ray Diffraction Lab, Memorial University of Newfoundland, St Johns, NL, Canada A1B 3X7

## Abstract

The title mol­ecule, C_58_H_44_N_6_S_8_, has point symmetry 2 (in the Schönfliess notation *C*_2_). The related crystallographic twofold axis bis­ects the central ethane bond while it is parallel to the monoclinic unique axis of the unit cell. The dithiole=C—C=dithiole torsion angle is 103.7 (4)° and the triazole–anthracene moieties adopt a pincer-like conformation. The crystal structure features C—H⋯S and C—H⋯N contacts. The distance between the stacked anthracene fragments [centroid—centroid separations of 3.6871 (19) Å, off-set by 1.516 (3) Å and mean anthracene plane-plane separations of 3.361 (2) Å], which are parallel to (101) and (-101), indicates inter­molecular anthracene–anthracene π–π contacts. One of the terminal methyl­sulfanyl groups was modelled as being disordered with two refined orientations that converged to occupancies of 0.809 (5) and 0.191 (5).

## Related literature

The simpler analogues, 4,4′,5,5′-tetra­methyl­thiol­ato-2,2′-ethane­diyl­idene(1,3-dithiole) and bis­(4,5-bis­(methyl­thio)-1,3-dithiol-2-yl­idene)succinonitrile, were previously reported by Bryce *et al.* (1996 ▶) and Jia *et al.* (2005 ▶), respectively. For information on the photophysical properties of tetra­thia­fulvalene vinyl­ogues, see: Mulla *et al.* (2012 ▶). For synthesis *via* Cu-catalysed alkyne–azide coupling reactions employed for the title compound, see: Meldal & Tornøe (2008 ▶); Hein *et al.* (2010 ▶); Zhao *et al.* (2012 ▶). For a discussion of hydrogen bonding, including non-traditional inter­actions, see: Desiraju (2011 ▶); Arunan *et al.* (2011 ▶). For standard bond lengths, see: Allen *et al.* (1987 ▶) and for a description of the Cambridge Structural Database, see Allen (2002 ▶).
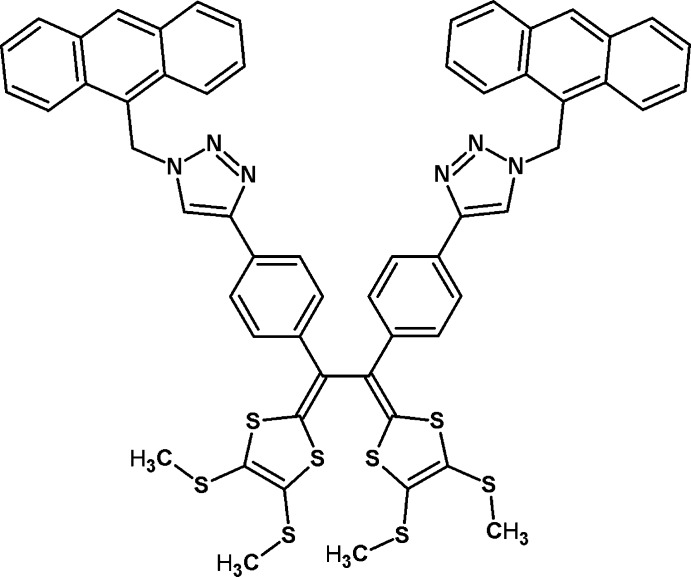


## Experimental

### 

#### Crystal data

C_58_H_44_N_6_S_8_
*M* *_r_* = 1081.47
Monoclinic, 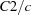

*a* = 15.906 (4) Å
*b* = 14.737 (3) Å
*c* = 21.570 (5) Å
β = 98.846 (7)°
*V* = 4996 (2) Å^3^
*Z* = 4
Mo *K*α radiation
μ = 0.41 mm^−1^
*T* = 168 K
0.27 × 0.23 × 0.12 mm

#### Data collection

Rigaku AFC8 diffractometer
Absorption correction: multi-scan (*REQAB*; Jacobson, 1998 ▶) *T*_min_ = 0.765, *T*_max_ = 0.952
24431 measured reflections
5169 independent reflections
4127 reflections with *I* > 2σ(*I*)
*R*_int_ = 0.059

#### Refinement

*R*[*F* ^2^ > 2σ(*F* ^2^)] = 0.058
*wR*(*F* ^2^) = 0.160
*S* = 1.06
5169 reflections
347 parameters
3 restraints
H-atom parameters constrained
Δρ_max_ = 0.70 e Å^−3^
Δρ_min_ = −0.62 e Å^−3^

### 

Data collection: *CrystalClear-SM Expert* (Rigaku, 2009 ▶); cell refinement: *CrystalClear-SM Expert*; data reduction: *CrystalClear-SM Expert*; program(s) used to solve structure: *SHELXS97* (Sheldrick, 2008 ▶); program(s) used to refine structure: *SHELXL97* (Sheldrick, 2008 ▶); molecular graphics: *Mercury* (Macrae *et al.*, 2006 ▶); software used to prepare material for publication: *OLEX2* (Dolomanov *et al.*, 2009 ▶), *publCIF* (Westrip, 2010 ▶) and *PLATON* (Spek, 2009 ▶).

## Supplementary Material

Crystal structure: contains datablock(s) I, global. DOI: 10.1107/S1600536812045254/fb2271sup1.cif

Structure factors: contains datablock(s) I. DOI: 10.1107/S1600536812045254/fb2271Isup2.hkl

Supplementary material file. DOI: 10.1107/S1600536812045254/fb2271Isup3.cml

Additional supplementary materials:  crystallographic information; 3D view; checkCIF report

## Figures and Tables

**Table 1:** Hydrogen-bond geometry (Å, °)

*D*—H⋯*A*	*D*—H	H⋯*A*	*D*⋯*A*	*D*—H⋯*A*
C28—H28*C*⋯N2^i^	0.98	2.55	3.495 (5)	161
C25—H25⋯S2^ii^	0.95	3.00	3.784 (4)	141

Symmetry codes: (i) 
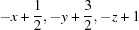
; (ii) 
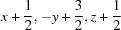
.

**Fig. 1.**
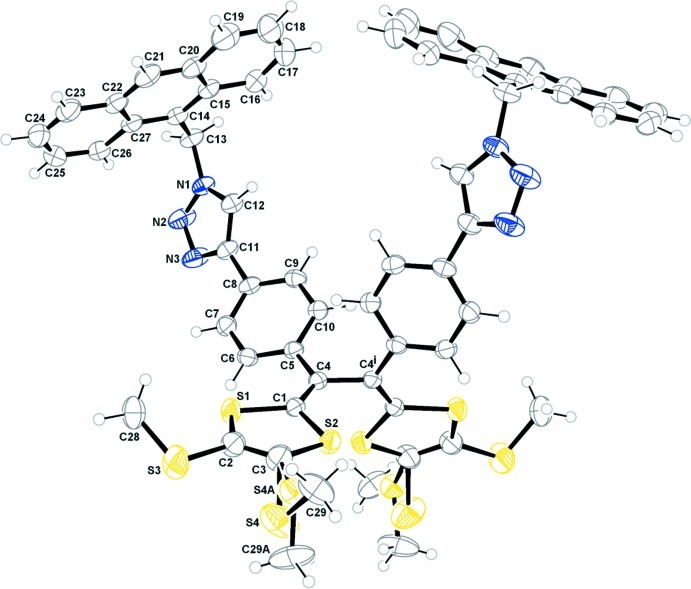
View of the title molecule. The displacement ellipsoids are shown at the 50% probability level. Symmetry code: (i) = -x, y, 1/2 - z.

**Fig. 2.**
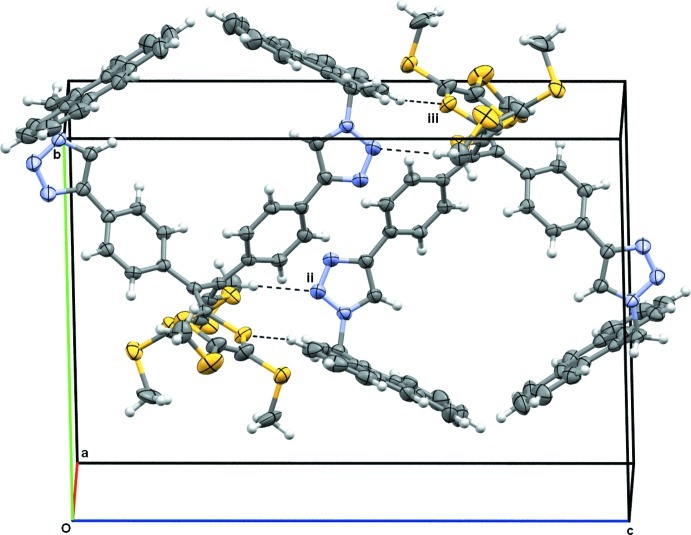
Short C—H···S and C—H···N contacts between the molecules of the title compound. Symmetry codes: (i) -x, y, 1/2 - z; (ii) 1/2 - x, 3/2 - y, 1 - z; (iii) 1/2 + x, 3/2 - y, 1/2 + z.

**Fig. 3.**
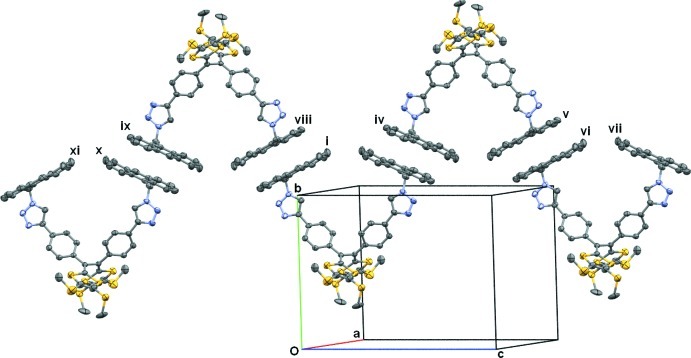
π-electron—π-electron interactions between the anthracene units, propagating parallel to (1, 0, 1), with the centroid-centroid separations of 3.6871 (19) Å (as measured between x, y, z and (iv) = 1/2 - x, 5/2 - y, 1 - z). Symmetry codes: (i) -x, y, 1/2 - z; (iv) 1/2 - x, 5/2 - y, 1 - z; (v) 1/2 + x, 5/2 - y, 1/2 + z; (vi) 1 - x, y, 3/2 - z; (vii) 1 + x, y, 1 + z; (viii) -1/2 + x, 5/2 - y, -1/2 + z; (ix) -1/2 - x, 5/2 - y, -z; (x) -1 + x, y, -1 + z; (xi) -1 - x, y, -1/2 - z.

## supplementary crystallographic information

### Comment

The asymmetric unit of the title molecule contains a half of the molecule. The whole molecule has the point symmetry 2 (or *C*_2_ in the Schönfliess notation). The twofold axis is parallel to the monoclinic axis and passes through the centre of the C4—C4^i^ bond (i = -*x*, *y*, 1/2 - *z*; Fig. 1). This point symmetry has been previously reported for the related molecule, bis(4,5-bis(methylthio)-1,3-dithiol-2-ylidene)succinonitrile (Jia *et al.* 2005). The dithiole═C—C═dithiole torsion angle is 103.7 (4)° for the title molecule, while for the related analogue Jia *et al.* (2005) reported 91.7° (no reported s.u.). In the Cambridge Crystallographic Database (Allen, 2002), the article by Jia *et al.* (2005) is deposited as CCDC 285669, refcode JAVWEJ, and the pertinent torsion angle has been given as 85.9 (7)°. The larger value of this torsion angle in the title molecule may result from the adoption of a pincer-like conformation of the triazole and anthracene fragments. Both the title molecule and that reported by Jia *et al.* (2005) exhibit a twisted *cis* conformation about the C4—C4^i^ bond (i = -*x*, *y*, 1/2 -*z*), which contrasts sharply with the planar 2,2'-ethanediylidene(1,3-dithiole) framework reported for 4,4',5,5'-tetramethylthiolato-2,2'-ethanediylidene(1,3-dithiole) (Bryce *et al.*, 1996), which adopts a *trans* conformation.

One of the terminal S—CH_3_ groups was disordered with two refined orientations that converged to occupancy 0.809 (5) for S4, C29 and the attached methyl H atoms H29A, H29B, H29C, and 0.191 (5) for S4A, C29A and the attached methyl H atoms H29D, H29E, H29F. (These hydrogens were difficult to discern in the difference map.)

Non-traditional D–H···A interactions (Arunan *et al.*, 2011, and Desiraju, 2011) are present betweent C25_anthracene_—H25···S2^ii^ ((ii) = 1/2 - *x*, 3/2 - *y*, 1 - *z*) and C28_methyl_–H28C···N2^iii^ ((iii) = *x* + 1/2, -*y* + 3/2, *z* + 1/2, see Fig. 2 and Table 1). Further intermolecular interactions in the form of π-electron _anthracene_—π-electron _anthracene_ contacts with centroid—centroid separations of 3.6871 (19) Å, off-set by 1.516 (3) Å and mean anthracene plane-plane separations of 3.361 (2) Å are present (Fig. 3; as measured from the anthracene fragment in *x*, *y*, *z* to (iv) = 1/2 - *x*, 5/2 - *y*, 1 - *z*). The molecules that exhibit H-bonding and π-electron—π-electron interactions are parallel to the (1, 0, 1) and (-1, 0, 1) planes (determined using *OLEX2* by Dolomanov *et al.*, 2009.)

### Experimental

The title compound was synthesized by the procedures reported by Mulla *et al.* (2012). 3 mg of the title compound were added to 0.8 ml of chloroform in a vial and heated to 323 K to give a clear solution. This solution was first cooled to room temperature and then methanol (0.5 ml) was added slowly to the chloroform solution by diffusion at 277 K over 3 days. After this time, yellow prismatic crystals with dimensions similar to those of the measured sample (0.27 × 0.23 × 0.12 mm), formed.

### Refinement

While all hydrogen atoms appeared in the difference electron density map, except those pertinent to the disordered groups, they were introduced into idealized positions and refined using the riding atom formalism. The applied constraints were: C_aryl_—H_aryl_ = 0.95 Å, C_methyl_—H_methyl_ = 0.98 Å, C_methylene_—H_methylene_ = 0.99 Å; *U*_iso_(H_aryl_) = 1.2*U*_eq_(C_aryl_), *U*_iso_(H_methylene_) = 1.2*U*_eq_(C_methylene_), *U*_iso_(H_methyl_) = 1.5*U*_eq_(C_methyl_). One terminal S—CH_3_ group was disordered with two orientations for S4—C29 (and the pertinent H-atoms) and S4A—C29A (and the pertinent H-atoms) the occupancies of which were constrained to equal to 1. (The respective occupancies resulted in 0.809 (5) and 0.191 (5).) A distance restraint (the command SADI from *SHELXL97* by Sheldrick, 2008) was applied to the S3—C28, S4—C29 and S4A—C29A bonds. The converged S—C bond lengths were consistent with those reported by Allen *et al.* (1987).

### Crystal data

**Table:**

C_58_H_44_N_6_S_8_	*F*(000) = 2248
*M**_r_* = 1081.47	*D*_x_ = 1.438 Mg m^−^^3^
Monoclinic, *C*2/*c*	Mo *K*α radiation, λ = 0.71075 Å
Hall symbol: -C 2yc	Cell parameters from 12368 reflections
*a* = 15.906 (4) Å	θ = 3.3–27.6°
*b* = 14.737 (3) Å	µ = 0.41 mm^−^^1^
*c* = 21.570 (5) Å	*T* = 168 K
β = 98.846 (7)°	Prism, yellow
*V* = 4996 (2) Å^3^	0.27 × 0.23 × 0.12 mm
*Z* = 4	

### Data collection

**Table:**

Rigaku AFC8 diffractometer	5169 independent reflections
Radiation source: fine-focus sealed tube	4127 reflections with *I* > 2σ(*I*)
Graphite - Rigaku SHINE monochromator	*R*_int_ = 0.059
Detector resolution: 14.63 pixels mm^-1^	θ_max_ = 26.5°, θ_min_ = 3.3°
ω scans	*h* = −19→19
Absorption correction: multi-scan (*REQAB*; Jacobson, 1998)	*k* = −18→18
*T*_min_ = 0.765, *T*_max_ = 0.952	*l* = −27→24
24431 measured reflections	

### Refinement

**Table:**

Refinement on *F*^2^	Primary atom site location: structure-invariant direct methods
Least-squares matrix: full	Secondary atom site location: difference Fourier map
*R*[*F*^2^ > 2σ(*F*^2^)] = 0.058	Hydrogen site location: difference Fourier map
*wR*(*F*^2^) = 0.160	H-atom parameters constrained
*S* = 1.06	*w* = 1/[σ^2^(*F*_o_^2^) + (0.0767*P*)^2^ + 8.5759*P*] where *P* = (*F*_o_^2^ + 2*F*_c_^2^)/3
5169 reflections	(Δ/σ)_max_ < 0.001
347 parameters	Δρ_max_ = 0.70 e Å^−^^3^
3 restraints	Δρ_min_ = −0.62 e Å^−^^3^
97 constraints	

### Special details

**Table:**

Geometry. All e.s.d.'s (except the e.s.d. in the dihedral angle between two l.s. planes) are estimated using the full covariance matrix. The cell e.s.d.'s are taken into account individually in the estimation of e.s.d.'s in distances, angles and torsion angles; correlations between e.s.d.'s in cell parameters are only used when they are defined by crystal symmetry. An approximate (isotropic) treatment of cell e.s.d.'s is used for estimating e.s.d.'s involving l.s. planes.
Refinement. Refinement of *F*^2^ against ALL reflections. The weighted *R*-factor *wR* and goodness of fit *S* are based on *F*^2^, conventional *R*-factors *R* are based on *F*, with *F* set to zero for negative *F*^2^. The threshold expression of *F*^2^ > σ(*F*^2^) is used only for calculating *R*-factors(gt) *etc*. and is not relevant to the choice of reflections for refinement. *R*-factors based on *F*^2^ are statistically about twice as large as those based on *F*, and *R*- factors based on ALL data will be even larger.

### Fractional atomic coordinates and isotropic or equivalent isotropic displacement parameters (Å^2^)

**Table:**

	*x*	*y*	*z*	*U*_iso_*/*U*_eq_	Occ. (<1)
S1	0.21077 (4)	0.55797 (5)	0.29159 (4)	0.0438 (2)	
S2	0.08541 (4)	0.49862 (5)	0.18534 (4)	0.0415 (2)	
S3	0.35601 (5)	0.46517 (8)	0.24249 (6)	0.0746 (3)	
S4	0.21209 (7)	0.41217 (8)	0.11540 (6)	0.0497 (5)	0.809 (5)
C29	0.2002 (4)	0.2963 (3)	0.1414 (3)	0.0846 (19)	0.809 (5)
H29A	0.2504	0.2791	0.1714	0.127*	0.809 (5)
H29B	0.1943	0.2554	0.1052	0.127*	0.809 (5)
H29C	0.1493	0.2919	0.1618	0.127*	0.809 (5)
S4A	0.2162 (5)	0.3602 (6)	0.1509 (3)	0.086 (3)	0.191 (5)
C29A	0.1999 (15)	0.4153 (16)	0.0740 (10)	0.073 (6)	0.191 (5)
H29D	0.1510	0.4564	0.0711	0.109*	0.191 (5)
H29E	0.1891	0.3691	0.0411	0.109*	0.191 (5)
H29F	0.2508	0.4500	0.0687	0.109*	0.191 (5)
N1	0.11476 (15)	1.01534 (15)	0.50733 (10)	0.0358 (5)	
N2	0.1323 (2)	0.95560 (17)	0.55383 (12)	0.0527 (7)	
N3	0.1189 (2)	0.87389 (17)	0.52924 (12)	0.0538 (7)	
C1	0.10424 (15)	0.56405 (17)	0.25385 (12)	0.0318 (5)	
C2	0.24892 (19)	0.4963 (2)	0.23200 (16)	0.0487 (8)	
C3	0.1915 (2)	0.4702 (2)	0.18276 (17)	0.0513 (8)	
C4	0.04180 (15)	0.61453 (16)	0.27167 (12)	0.0289 (5)	
C5	0.05131 (15)	0.67558 (16)	0.32632 (12)	0.0297 (5)	
C6	0.11164 (16)	0.66476 (17)	0.38029 (12)	0.0343 (6)	
H6	0.1458	0.6115	0.3846	0.041*	
C7	0.12316 (17)	0.72875 (18)	0.42735 (13)	0.0366 (6)	
H7	0.1642	0.7188	0.4636	0.044*	
C8	0.07492 (17)	0.80792 (17)	0.42201 (12)	0.0339 (6)	
C9	0.01149 (17)	0.81784 (17)	0.37050 (13)	0.0346 (6)	
H9	−0.0235	0.8704	0.3671	0.041*	
C10	−0.00144 (16)	0.75248 (17)	0.32412 (12)	0.0319 (5)	
H10	−0.0467	0.7596	0.2902	0.038*	
C11	0.09216 (17)	0.88302 (18)	0.46645 (12)	0.0360 (6)	
C12	0.08963 (17)	0.97300 (18)	0.45269 (13)	0.0371 (6)	
H12	0.0734	1.0002	0.4127	0.045*	
C13	0.13241 (18)	1.11193 (18)	0.51750 (14)	0.0390 (6)	
H13A	0.1583	1.1224	0.5617	0.047*	
H13B	0.0787	1.1468	0.5092	0.047*	
C14	0.19234 (17)	1.14373 (17)	0.47421 (12)	0.0336 (6)	
C15	0.16101 (18)	1.18757 (18)	0.41722 (13)	0.0383 (6)	
C16	0.0730 (2)	1.2060 (2)	0.39650 (16)	0.0506 (8)	
H16	0.0316	1.1858	0.4207	0.061*	
C17	0.0483 (3)	1.2525 (3)	0.34189 (19)	0.0704 (11)	
H17	−0.0105	1.2639	0.3289	0.085*	
C18	0.1066 (3)	1.2840 (3)	0.30463 (17)	0.0723 (11)	
H18	0.0878	1.3185	0.2679	0.087*	
C19	0.1897 (3)	1.2648 (3)	0.32130 (15)	0.0641 (10)	
H19	0.2291	1.2845	0.2952	0.077*	
C20	0.2200 (2)	1.2156 (2)	0.37696 (13)	0.0434 (7)	
C21	0.3054 (2)	1.1964 (2)	0.39396 (14)	0.0457 (7)	
H21	0.3440	1.2136	0.3665	0.055*	
C22	0.33685 (18)	1.15312 (18)	0.44937 (14)	0.0408 (7)	
C23	0.4252 (2)	1.1359 (2)	0.46688 (17)	0.0510 (8)	
H23	0.4636	1.1531	0.4393	0.061*	
C24	0.4557 (2)	1.0955 (2)	0.5221 (2)	0.0609 (10)	
H24	0.5152	1.0864	0.5337	0.073*	
C25	0.3994 (2)	1.0672 (2)	0.56234 (18)	0.0578 (9)	
H25	0.4211	1.0381	0.6008	0.069*	
C26	0.3150 (2)	1.08074 (19)	0.54724 (15)	0.0459 (7)	
H26	0.2783	1.0599	0.5750	0.055*	
C27	0.27920 (17)	1.12572 (17)	0.49037 (13)	0.0359 (6)	
C28	0.40626 (19)	0.5551 (3)	0.29117 (17)	0.0573 (9)	
H28A	0.3965	0.6131	0.2689	0.086*	
H28B	0.4675	0.5435	0.3008	0.086*	
H28C	0.3821	0.5578	0.3303	0.086*	

### Atomic displacement parameters (Å^2^)

**Table:**

	*U*^11^	*U*^22^	*U*^33^	*U*^12^	*U*^13^	*U*^23^
S1	0.0279 (3)	0.0485 (4)	0.0533 (5)	0.0072 (3)	0.0005 (3)	−0.0139 (3)
S2	0.0340 (4)	0.0417 (4)	0.0479 (4)	0.0033 (3)	0.0030 (3)	−0.0147 (3)
S3	0.0349 (4)	0.0816 (7)	0.1068 (8)	0.0138 (4)	0.0091 (5)	−0.0313 (6)
S4	0.0565 (7)	0.0525 (8)	0.0425 (9)	0.0104 (5)	0.0157 (5)	−0.0084 (7)
C29	0.121 (5)	0.041 (3)	0.106 (4)	−0.009 (3)	0.061 (4)	−0.033 (3)
S4A	0.108 (5)	0.083 (6)	0.070 (5)	0.044 (4)	0.023 (3)	−0.014 (4)
C29A	0.085 (16)	0.069 (14)	0.067 (16)	0.022 (11)	0.020 (13)	0.023 (13)
N1	0.0426 (12)	0.0321 (11)	0.0351 (12)	−0.0097 (10)	0.0138 (10)	−0.0043 (9)
N2	0.082 (2)	0.0406 (14)	0.0353 (14)	−0.0204 (13)	0.0083 (13)	−0.0019 (11)
N3	0.084 (2)	0.0374 (13)	0.0388 (14)	−0.0203 (13)	0.0053 (13)	0.0008 (11)
C1	0.0282 (12)	0.0273 (12)	0.0395 (14)	−0.0009 (10)	0.0040 (11)	−0.0022 (10)
C2	0.0343 (14)	0.0473 (17)	0.066 (2)	0.0055 (13)	0.0109 (14)	−0.0130 (15)
C3	0.0421 (16)	0.0500 (18)	0.063 (2)	0.0038 (14)	0.0131 (15)	−0.0183 (15)
C4	0.0271 (12)	0.0248 (11)	0.0347 (13)	−0.0013 (9)	0.0046 (10)	0.0015 (10)
C5	0.0296 (12)	0.0255 (12)	0.0349 (13)	−0.0025 (10)	0.0080 (10)	−0.0010 (10)
C6	0.0341 (13)	0.0277 (12)	0.0413 (15)	0.0017 (10)	0.0068 (11)	0.0019 (11)
C7	0.0363 (14)	0.0360 (14)	0.0371 (14)	−0.0040 (11)	0.0036 (11)	0.0009 (11)
C8	0.0383 (14)	0.0285 (12)	0.0371 (14)	−0.0082 (11)	0.0127 (11)	−0.0016 (11)
C9	0.0363 (13)	0.0282 (12)	0.0413 (15)	0.0027 (10)	0.0127 (11)	−0.0002 (11)
C10	0.0301 (12)	0.0320 (13)	0.0348 (13)	0.0002 (10)	0.0085 (10)	−0.0002 (11)
C11	0.0380 (14)	0.0347 (13)	0.0371 (15)	−0.0084 (11)	0.0115 (11)	−0.0029 (11)
C12	0.0422 (15)	0.0350 (14)	0.0353 (14)	−0.0058 (12)	0.0095 (12)	−0.0019 (11)
C13	0.0443 (15)	0.0311 (13)	0.0447 (16)	−0.0080 (12)	0.0166 (13)	−0.0090 (12)
C14	0.0378 (13)	0.0269 (12)	0.0377 (14)	−0.0062 (10)	0.0104 (11)	−0.0053 (11)
C15	0.0425 (15)	0.0304 (13)	0.0417 (15)	−0.0071 (11)	0.0057 (12)	−0.0076 (11)
C16	0.0450 (17)	0.0441 (17)	0.060 (2)	−0.0063 (13)	−0.0006 (15)	−0.0017 (15)
C17	0.061 (2)	0.067 (2)	0.073 (3)	−0.0036 (19)	−0.023 (2)	0.003 (2)
C18	0.086 (3)	0.078 (3)	0.046 (2)	−0.015 (2)	−0.014 (2)	0.0145 (19)
C19	0.086 (3)	0.065 (2)	0.0386 (18)	−0.023 (2)	0.0020 (17)	0.0035 (16)
C20	0.0565 (18)	0.0392 (15)	0.0349 (15)	−0.0153 (13)	0.0080 (13)	−0.0058 (12)
C21	0.0510 (17)	0.0458 (16)	0.0440 (16)	−0.0165 (14)	0.0194 (14)	−0.0092 (13)
C22	0.0410 (15)	0.0325 (13)	0.0512 (17)	−0.0075 (12)	0.0143 (13)	−0.0109 (13)
C23	0.0403 (16)	0.0421 (16)	0.074 (2)	−0.0066 (13)	0.0198 (16)	−0.0111 (16)
C24	0.0382 (16)	0.0437 (18)	0.099 (3)	0.0002 (14)	0.0030 (18)	−0.0094 (19)
C25	0.0546 (19)	0.0392 (16)	0.074 (2)	0.0006 (15)	−0.0084 (17)	0.0083 (16)
C26	0.0473 (16)	0.0346 (14)	0.0541 (18)	−0.0089 (13)	0.0024 (14)	0.0052 (13)
C27	0.0383 (14)	0.0261 (12)	0.0444 (15)	−0.0062 (11)	0.0096 (12)	−0.0051 (11)
C28	0.0326 (15)	0.079 (2)	0.060 (2)	0.0004 (15)	0.0090 (14)	−0.0008 (18)

### Geometric parameters (Å, º)

**Table:**

S1—C2	1.757 (3)	C10—H10	0.9500
S1—C1	1.766 (3)	C11—C12	1.358 (4)
S2—C3	1.747 (3)	C12—H12	0.9500
S2—C1	1.751 (3)	C13—C14	1.508 (4)
S3—C2	1.745 (3)	C13—H13A	0.9900
S3—C28	1.800 (4)	C13—H13B	0.9900
S4—C3	1.760 (3)	C14—C27	1.397 (4)
S4—C29	1.817 (5)	C14—C15	1.410 (4)
C29—H29A	0.9800	C15—C16	1.428 (4)
C29—H29B	0.9800	C15—C20	1.434 (4)
C29—H29C	0.9800	C16—C17	1.367 (5)
S4A—C3	1.827 (7)	C16—H16	0.9500
S4A—C29A	1.829 (16)	C17—C18	1.396 (6)
C29A—H29D	0.9800	C17—H17	0.9500
C29A—H29E	0.9800	C18—C19	1.345 (6)
C29A—H29F	0.9800	C18—H18	0.9500
N1—N2	1.332 (3)	C19—C20	1.421 (5)
N1—C12	1.339 (3)	C19—H19	0.9500
N1—C13	1.461 (3)	C20—C21	1.382 (4)
N2—N3	1.320 (3)	C21—C22	1.379 (4)
N3—C11	1.362 (4)	C21—H21	0.9500
C1—C4	1.343 (3)	C22—C23	1.421 (4)
C2—C3	1.347 (5)	C22—C27	1.427 (4)
C4—C5	1.472 (3)	C23—C24	1.353 (5)
C4—C4^i^	1.503 (5)	C23—H23	0.9500
C5—C6	1.399 (4)	C24—C25	1.403 (5)
C5—C10	1.406 (3)	C24—H24	0.9500
C6—C7	1.377 (4)	C25—C26	1.346 (4)
C6—H6	0.9500	C25—H25	0.9500
C7—C8	1.391 (4)	C26—C27	1.433 (4)
C7—H7	0.9500	C26—H26	0.9500
C8—C9	1.389 (4)	C28—H28A	0.9800
C8—C11	1.462 (4)	C28—H28B	0.9800
C9—C10	1.381 (4)	C28—H28C	0.9800
C9—H9	0.9500		
			
C2—S1—C1	95.96 (14)	N1—C13—C14	109.6 (2)
C3—S2—C1	96.79 (14)	N1—C13—H13A	109.8
C2—S3—C28	102.65 (15)	C14—C13—H13A	109.8
C3—S4—C29	99.2 (2)	N1—C13—H13B	109.8
C3—S4A—C29A	86.5 (9)	C14—C13—H13B	109.8
S4A—C29A—H29D	109.5	H13A—C13—H13B	108.2
S4A—C29A—H29E	109.5	C27—C14—C15	120.5 (2)
H29D—C29A—H29E	109.5	C27—C14—C13	118.8 (3)
S4A—C29A—H29F	109.5	C15—C14—C13	120.6 (3)
H29D—C29A—H29F	109.5	C14—C15—C16	123.9 (3)
H29E—C29A—H29F	109.5	C14—C15—C20	118.9 (3)
N2—N1—C12	110.8 (2)	C16—C15—C20	117.2 (3)
N2—N1—C13	121.2 (2)	C17—C16—C15	120.2 (3)
C12—N1—C13	127.6 (2)	C17—C16—H16	119.9
N3—N2—N1	107.3 (2)	C15—C16—H16	119.9
N2—N3—C11	108.4 (2)	C16—C17—C18	122.3 (4)
C4—C1—S2	120.55 (19)	C16—C17—H17	118.9
C4—C1—S1	126.5 (2)	C18—C17—H17	118.9
S2—C1—S1	112.87 (14)	C19—C18—C17	119.3 (3)
C3—C2—S3	123.9 (2)	C19—C18—H18	120.4
C3—C2—S1	117.1 (2)	C17—C18—H18	120.4
S3—C2—S1	118.77 (19)	C18—C19—C20	121.6 (4)
C2—C3—S2	116.6 (2)	C18—C19—H19	119.2
C2—C3—S4	126.9 (2)	C20—C19—H19	119.2
S2—C3—S4	116.51 (19)	C21—C20—C19	121.4 (3)
C2—C3—S4A	113.0 (3)	C21—C20—C15	119.2 (3)
S2—C3—S4A	119.3 (3)	C19—C20—C15	119.4 (3)
C1—C4—C5	125.3 (2)	C22—C21—C20	122.4 (3)
C1—C4—C4^i^	116.4 (2)	C22—C21—H21	118.8
C5—C4—C4^i^	118.0 (2)	C20—C21—H21	118.8
C6—C5—C10	116.7 (2)	C21—C22—C23	121.6 (3)
C6—C5—C4	124.7 (2)	C21—C22—C27	119.1 (3)
C10—C5—C4	118.6 (2)	C23—C22—C27	119.2 (3)
C7—C6—C5	122.1 (2)	C24—C23—C22	121.2 (3)
C7—C6—H6	118.9	C24—C23—H23	119.4
C5—C6—H6	118.9	C22—C23—H23	119.4
C6—C7—C8	120.3 (3)	C23—C24—C25	119.9 (3)
C6—C7—H7	119.9	C23—C24—H24	120.0
C8—C7—H7	119.9	C25—C24—H24	120.0
C9—C8—C7	118.5 (2)	C26—C25—C24	120.9 (3)
C9—C8—C11	119.0 (2)	C26—C25—H25	119.5
C7—C8—C11	122.3 (3)	C24—C25—H25	119.5
C10—C9—C8	121.0 (2)	C25—C26—C27	121.7 (3)
C10—C9—H9	119.5	C25—C26—H26	119.1
C8—C9—H9	119.5	C27—C26—H26	119.1
C9—C10—C5	121.1 (2)	C14—C27—C22	119.8 (3)
C9—C10—H10	119.5	C14—C27—C26	123.3 (3)
C5—C10—H10	119.5	C22—C27—C26	116.9 (3)
C12—C11—N3	107.9 (2)	S3—C28—H28A	109.5
C12—C11—C8	126.8 (3)	S3—C28—H28B	109.5
N3—C11—C8	125.1 (2)	H28A—C28—H28B	109.5
N1—C12—C11	105.5 (2)	S3—C28—H28C	109.5
N1—C12—H12	127.2	H28A—C28—H28C	109.5
C11—C12—H12	127.2	H28B—C28—H28C	109.5
			
C12—N1—N2—N3	0.3 (3)	C9—C8—C11—C12	−36.6 (4)
C13—N1—N2—N3	−172.9 (3)	C7—C8—C11—C12	139.4 (3)
N1—N2—N3—C11	−0.3 (4)	C9—C8—C11—N3	147.6 (3)
C3—S2—C1—C4	169.8 (2)	C7—C8—C11—N3	−36.3 (4)
C3—S2—C1—S1	−7.97 (19)	N2—N1—C12—C11	−0.2 (3)
C2—S1—C1—C4	−170.3 (3)	C13—N1—C12—C11	172.5 (3)
C2—S1—C1—S2	7.28 (18)	N3—C11—C12—N1	0.0 (3)
C28—S3—C2—C3	155.2 (3)	C8—C11—C12—N1	−176.4 (3)
C28—S3—C2—S1	−30.0 (3)	N2—N1—C13—C14	121.3 (3)
C1—S1—C2—C3	−3.6 (3)	C12—N1—C13—C14	−50.6 (4)
C1—S1—C2—S3	−178.7 (2)	N1—C13—C14—C27	−79.6 (3)
S3—C2—C3—S2	173.3 (2)	N1—C13—C14—C15	97.9 (3)
S1—C2—C3—S2	−1.5 (4)	C27—C14—C15—C16	178.1 (3)
S3—C2—C3—S4	−7.9 (5)	C13—C14—C15—C16	0.7 (4)
S1—C2—C3—S4	177.2 (2)	C27—C14—C15—C20	−1.7 (4)
S3—C2—C3—S4A	29.4 (5)	C13—C14—C15—C20	−179.1 (2)
S1—C2—C3—S4A	−145.4 (4)	C14—C15—C16—C17	177.0 (3)
C1—S2—C3—C2	5.9 (3)	C20—C15—C16—C17	−3.2 (4)
C1—S2—C3—S4	−173.1 (2)	C15—C16—C17—C18	−0.2 (6)
C1—S2—C3—S4A	147.4 (4)	C16—C17—C18—C19	2.9 (6)
C29—S4—C3—C2	91.4 (4)	C17—C18—C19—C20	−2.0 (6)
C29—S4—C3—S2	−89.8 (3)	C18—C19—C20—C21	−179.8 (3)
C29—S4—C3—S4A	14.2 (5)	C18—C19—C20—C15	−1.5 (5)
C29A—S4A—C3—C2	−130.5 (9)	C14—C15—C20—C21	2.1 (4)
C29A—S4A—C3—S2	86.7 (9)	C16—C15—C20—C21	−177.7 (3)
C29A—S4A—C3—S4	−8.5 (8)	C14—C15—C20—C19	−176.2 (3)
S2—C1—C4—C5	−176.68 (19)	C16—C15—C20—C19	4.0 (4)
S1—C1—C4—C5	0.7 (4)	C19—C20—C21—C22	176.5 (3)
S2—C1—C4—C4^i^	−2.8 (3)	C15—C20—C21—C22	−1.8 (4)
S1—C1—C4—C4^i^	174.61 (18)	C20—C21—C22—C23	−178.4 (3)
C1—C4—C5—C6	−27.2 (4)	C20—C21—C22—C27	1.0 (4)
C4^i^—C4—C5—C6	159.0 (2)	C21—C22—C23—C24	178.2 (3)
C1—C4—C5—C10	150.5 (3)	C27—C22—C23—C24	−1.2 (4)
C4^i^—C4—C5—C10	−23.3 (3)	C22—C23—C24—C25	2.1 (5)
C10—C5—C6—C7	−4.2 (4)	C23—C24—C25—C26	−0.9 (5)
C4—C5—C6—C7	173.5 (2)	C24—C25—C26—C27	−1.1 (5)
C5—C6—C7—C8	−0.8 (4)	C15—C14—C27—C22	1.0 (4)
C6—C7—C8—C9	4.3 (4)	C13—C14—C27—C22	178.4 (2)
C6—C7—C8—C11	−171.8 (2)	C15—C14—C27—C26	−179.4 (2)
C7—C8—C9—C10	−2.6 (4)	C13—C14—C27—C26	−2.0 (4)
C11—C8—C9—C10	173.6 (2)	C21—C22—C27—C14	−0.6 (4)
C8—C9—C10—C5	−2.5 (4)	C23—C22—C27—C14	178.8 (2)
C6—C5—C10—C9	5.8 (4)	C21—C22—C27—C26	179.8 (3)
C4—C5—C10—C9	−172.0 (2)	C23—C22—C27—C26	−0.8 (4)
N2—N3—C11—C12	0.2 (4)	C25—C26—C27—C14	−177.7 (3)
N2—N3—C11—C8	176.6 (3)	C25—C26—C27—C22	1.9 (4)

Symmetry code: (i) −*x*, *y*, −*z*+1/2.

### Hydrogen-bond geometry (Å, º)

**Table:**

*D*—H···*A*	*D*—H	H···*A*	*D*···*A*	*D*—H···*A*
C28—H28*C*···N2^ii^	0.98	2.55	3.495 (5)	161
C25—H25···S2^iii^	0.95	3.00	3.784 (4)	141

Symmetry codes: (ii) −*x*+1/2, −*y*+3/2, −*z*+1; (iii) *x*+1/2, −*y*+3/2, *z*+1/2.
